# Cigarette Smoke During Breastfeeding in Rats Changes Glucocorticoid and Vitamin D Status in Obese Adult Offspring

**DOI:** 10.3390/ijms19103084

**Published:** 2018-10-09

**Authors:** Patricia Novaes Soares, Vanessa Silva Tavares Rodrigues, Thamara Cherem Peixoto, Camila Calvino, Rosiane Aparecida Miranda, Bruna Pereira Lopes, Nayara Peixoto-Silva, Luciana Lopes Costa, Sylvio Claudio-Neto, Alex Christian Manhães, Elaine Oliveira, Egberto Gaspar de Moura, Patricia Cristina Lisboa

**Affiliations:** 1 Laboratory of Endocrine Physiology, Biology Institute, State University of Rio de Janeiro, Rio de Janeiro, RJ 20551-031, Brazil; patriciasoares@uerj.br (P.N.S.); vanessa@uerj.br (V.S.T.R.); cherempeixoto@uerj.br (T.C.P.); calvino@uerj.br (C.C.); rosianemiranda@uerj.br (R.A.M.); brunalopes@uerj.br (B.P.L.); peixotosilva@uerj.br (N.P.-S.); lucianacosta@uerj.br (L.L.C.); elainedeoliveir@yahoo.com.br (E.O.); egbertogmoura@sr2.uerj.br (E.G.d.M.); 2 Laboratory of Neurophysiology, Biology Institute, State University of Rio de Janeiro, Rio de Janeiro, RJ 20551-031, Brazil; claudioneto@uerj.br (S.C.-N.); alexmanhaes@uerj.br (A.C.M.)

**Keywords:** cigarette smoke, lactation, programming, adipose tissue, liver

## Abstract

Maternal smoking increases obesogenesis in the progeny. Obesity is associated with several hormonal dysfunctions. In a rat model of postnatal tobacco smoke exposure, we previously reported increased central fat depot and disruption of some hormonal systems in the adult offspring. As both glucocorticoids and vitamin D alter lipogenesis and adipogenesis, here we evaluated the metabolism of these two hormones in visceral adipose tissue (VAT) and liver by Western blotting, and possible associations with lipogenesis biomarkers in adult rats that were exposed to tobacco smoke during their suckling period. At postnatal day (PN) 3, dams and offspring of both sexes were exposed (S group) or not (C group) to tobacco smoke, 4 × 1 h/day. At PN180, corticosteronemia was lower in S male and higher in S female offspring, without alterations in peripheral glucocorticoid metabolism and receptor. Adrenal ACTH receptor (MC2R) was higher in both sexes of S group. Despite unchanged serum vitamin D, liver 25-hydroxylase was higher in both sexes of S group. Male S offspring had higher 1α-hydroxylase, acetyl-CoA carboxylase (ACC), and fatty acid synthase (FAS) in VAT. Both sexes showed increased ACC protein content and reduced sirtuin mRNA in liver. Male S offspring had lower liver peroxisome proliferator-activated receptor-α. Tobacco exposure during lactation induced abdominal obesity in both sexes via distinct mechanisms. Males and females seem to develop HPA-axis dysfunction instead of changes in glucocorticoid metabolism and action. Lipogenesis in VAT and liver, as well as vitamin D status, are more influenced by postnatal smoke exposure in male than in female adult rat offspring.

## 1. Introduction

It is well established that smoking is a risk factor for the development of some chronic non-communicable diseases, such as respiratory diseases, some types of cancers, cardiovascular diseases, and glucose intolerance, in addition to being one of the major causes of early death worldwide [1,2]. Nowadays, about 1 billion people around the world consume tobacco, of which the consumption is responsible for the deaths of 6 million/year users [3]. According to the World Health Organization (WHO), this number will increase to 10 million by 2030, with 70% of those deaths occurring in developing countries [4]. The health problems caused by cigarette consumption are due to the large amounts of nocive chemicals that cigarettes contain, which almost doubles after burning [5]. Among these thousands of chemical compounds, nicotine (main psychoactive component), hydrocarbons, carbon monoxide, fungicides, pesticides, and hundreds of additives, as well as heavy metals, can be highlighted [6]. 

Even all the knowledge that has been thus far amassed regarding the health issues linked to cigarette consumption has not been sufficient to significantly reduce the number of users. In fact, smoking prevalence is still increasing in women, a particularly worrisome finding when one considers that during the critical period of gestation and lactation, the use of tobacco not only has negative consequences for the health of the mother, but also for that of the baby, causing difficulties in development as well as increasing neonatal and infant mortality [7,8]. Although pregnant women usually stop or at least reduce smoking during pregnancy, many of them resume the habit during breastfeeding [9,10], thereby exposing their children to some all the harmful substances present in the cigarette, whether through passive smoke or via breast milk. 

Several research groups have been studying the phenomenon of metabolic programming, also known as ontogenetic plasticity. It consists of epigenetic changes that occur in critical stages of development, such as gestation and lactation, which may increase the risk for the development, in adulthood, of diseases such as obesity, which is a serious public health problem of pandemic proportions [5,11,12]. Epidemiological studies evidenced the relationship between maternal smoking and the development of obesity in their children [13,14,15]. Rats are used in one of the programming animal models for which the outcome of tobacco smoke exposure during lactation is obesity. Our group has already shown that adult offspring of both sexes have hyperphagia, higher body mass, visceral fat, dyslipidemia, and hyperleptinemia [16,17], as well as other important endocrine disorders. Curiously, we have detected a sexual dimorphism for some hormones in this model: while males exhibit secondary hyperthyroidism, hypocorticosteronemia, and reduction of adrenal catecholamines [16], females show only higher T4 levels and hypercorticosteronemia [17].

Obesity is a chronic, multifactorial and pro-inflammatory disease, defined as a disproportionate increase in body mass generated by excessive fat accumulation in the adipose tissue [12]. This dysfunction occurs via two distinct processes that may or may not happen concomitantly: (a) hyperplasia (increase in the number of adipocytes); and (b) hypertrophy (increase in adipocyte area) [18]. Numerous factors act on these two processes; among them, hormones such as glucocorticoids (GC) and vitamin D. These steroid hormones act through binding to their specific receptors, which are members of the nuclear receptor superfamily of transcription factors, respectively GR (glucocorticoids receptor) and VDR (vitamin D receptor); both of them are important regulators of lipid metabolism [19,20,21].

Higher levels of GC plus higher tissue responsiveness to GC have both adipogenic and lipogenic effects [22,23]. GC excess results in pre-adipocyte differentiation and increases visceral fat accumulation [24,25]. Also, the hyperactivity of 11β-hydroxysteroid dehydrogenase 1 (11βHSD1), an enzyme that activates 11-dehydrocorticosterone into corticosterone to supply local GC demand, can increase adipose tissue mass [24,26,27]. In addition to its action on white adipocytes, GC also acts on lipogenesis in the liver, and is directly associated with the development of non-alcoholic fatty liver disease (NAFLD). In fact, patients with Cushing’s syndrome often have NAFLD [28,29].

Although vitamin D has a well known effect on the bone, some data have shown that this hormone has non-classic actions in the adipose tissue function that are particularly relevant for the development of obesity [30]. Some studies have already reported that vitamin D has a direct anti-adipogenic role [31,32,33]. In fact, the existence of a relationship between lower serum vitamin D levels and higher body mass has been suggested [34,35]. Contrasting with human studies, obese rodents show higher serum 25(OH)D levels associated with increased fat mass [36,37]. Furthermore, higher 1-alpha-hydroxylase (the enzyme that converts 25(OH)D to 1,25(OH)2D) in the visceral adipocyte and a lower VDR, have been reported, suggesting vitamin D resistance in the adipocyte [36,37]. Thus, the absence of vitamin D action could be obesogenic. Concerning liver tissue, epidemiological studies have identified an association between low levels of vitamin D and NAFLD [38,39]. In animals, vitamin D improves hepatic steatosis by preventing autophagy and oxidative stress [40,41], but the mechanism by which vitamin D attenuates NAFLD remains unclear.

Based on the effects of glucocorticoids and vitamin D on lipogenesis and on the phenotype shown by adult animals of both sexes that were programmed by neonatal exposure to cigarette smoke, the objective of the present study was to evaluate the metabolism of these two hormones and the their relationship with lipogenic markers in adipose tissue and liver of rats that were tobacco smoke-exposed from birth to weaning. We hypothesized that the metabolism and/or action of glucocorticoids and vitamin D in adipocytes and hepatocytes would be altered, and that these alterations could contribute to obesogenesis in these animals. Additionally, because sirtuin 1, a histone deacetylase that is associated with lower adiposity and protection against metabolic disorders [42,43], is inhibited by smoking [44], we also evaluated its mRNA expression in the liver to further elucidate possible molecular mechanisms.

## 2. Results

### 2.1. Histology and Lipid Content

**Figure 1:**
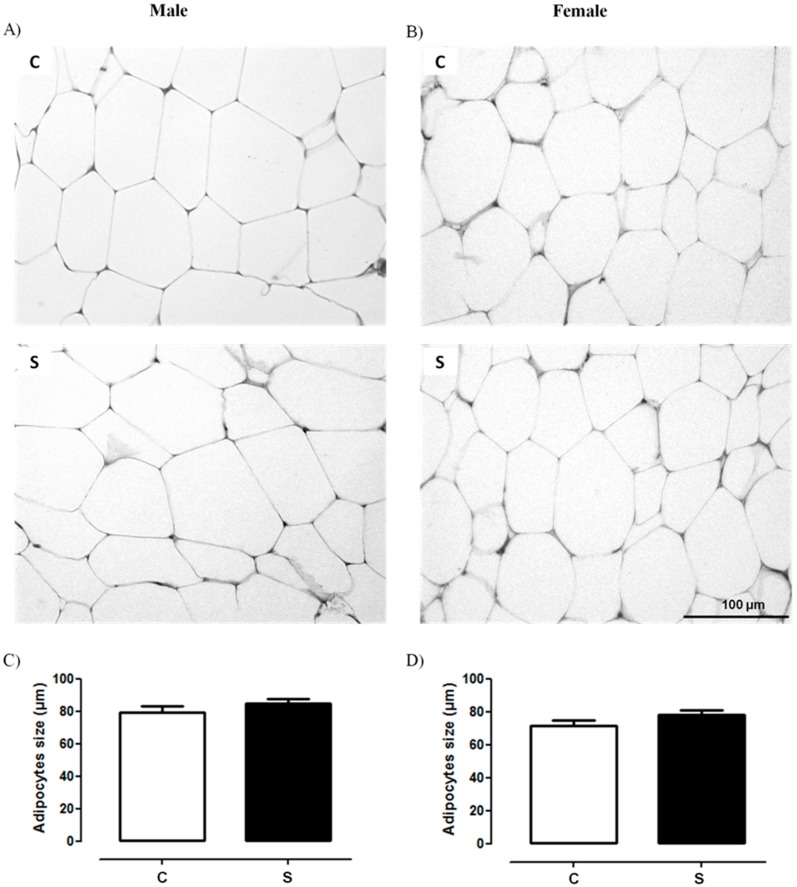
Representative photomicrographs (×40; hematoxylin-eosin) of the visceral adipose tissue of adult rats programmed by smoke exposure during the lactation. (**A**) morphology of C and S males; (**B**) morphology of C and S females; (**C**) retroperitoneal adipocyte area size of C and S males; (**D**) retroperitoneal adipocyte area size of C and S females. Both groups showed standard morphology, with no changes in the adipocyte area. Groups: C—CONTROL and S—SMOKE (*n* = 6 rats/group).

**Figure 2:**
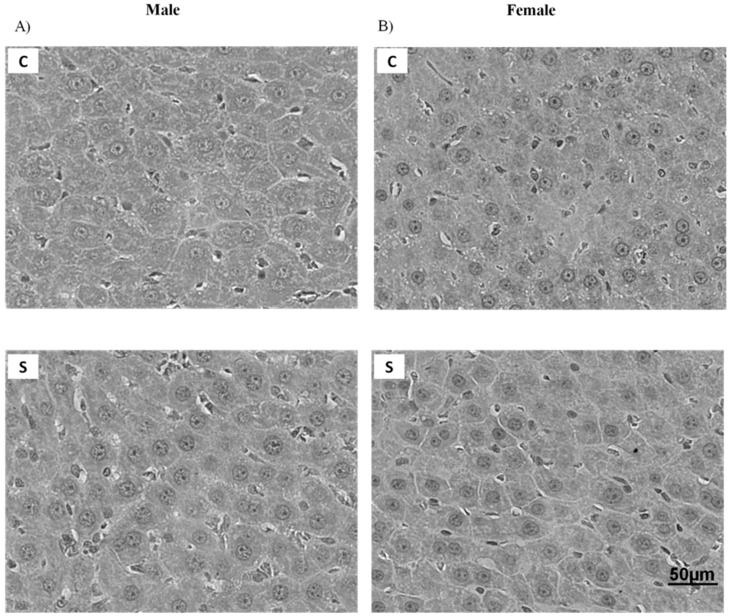
Representative photomicrographs (×60; hematoxylin-eosin) of the liver of adult rats programmed by smoke exposure during the lactation period. (**A**) morphology of C and S males; (**B**) morphology of C and S females. Both groups presented typical architecture, with no inflammatory cell infiltrate or drops of lipids in their cytoplasm. Groups: C—CONTROL and S—SMOKE (*n* = 6 rats/group).

Figure 1A,B and Figure 2 depict the morphology of retroperitoneal adipocytes and liver, respectively. C and S groups of both sexes showed preserved architectures of these tissues. Figure 1C,D show that visceral adipocyte size did not differ between the groups. According to the Brunt’s NAFLD score, hepatic histological evaluation of males and females of both groups showed grade 0, which means that <5% of hepatocytes were affected (Table 1). As shown in Table 2, there were no differences between S and C groups of both sexes regarding triglyceride and cholesterol levels in the liver.

### 2.2. Serum Corticosterone and Adrenal ACTH Receptor (MC2R).

**Figure 3:**
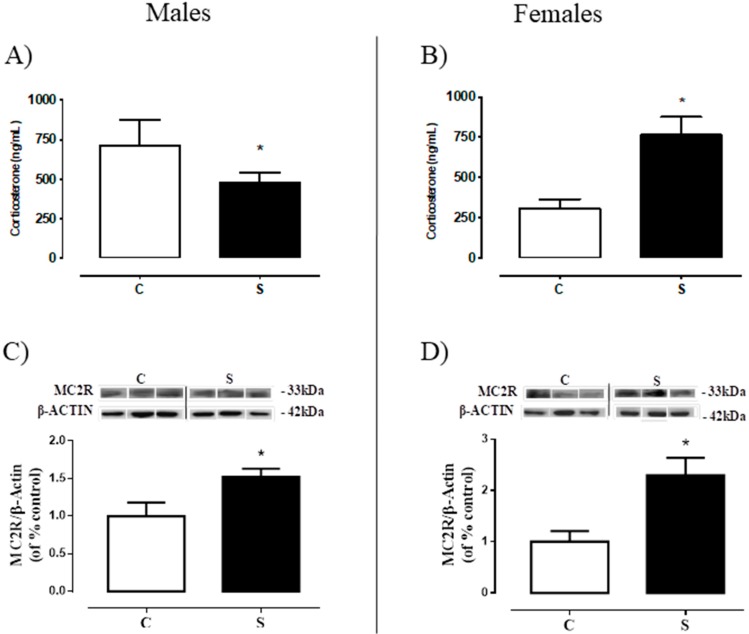
Corticosterone and ACTH receptor content in adult rats programmed by smoke exposure during the lactation period. Corticosterone levels in the plasma (**A**—male; **B**—female) and melanocortin 2 receptor (MC2R) content in the adrenal gland (**C**—male; **D**—female). Groups: C—CONTROL and S—SMOKE. alues are given as means ± S.E.M. of 8–10 rats/group. * *p* < 0.05 significant difference between S vs. C.

Male offspring of the S group showed lower corticosteronemia (−33% vs. C, *p* < 0.05, Figure 3A) and higher MC2R content in the adrenal gland (+52% vs. C, *p* < 0.05, Figure 3C), while S females had higher corticosteronemia and higher MC2R content (+1.5-fold vs. C, *p* < 0.05, Figure 3B; +1.3-fold vs. C, *p* < 0.05, Figure 3D; respectively).

### 2.3. Glucocorticoid Metabolism and Action

**Figure 4:**
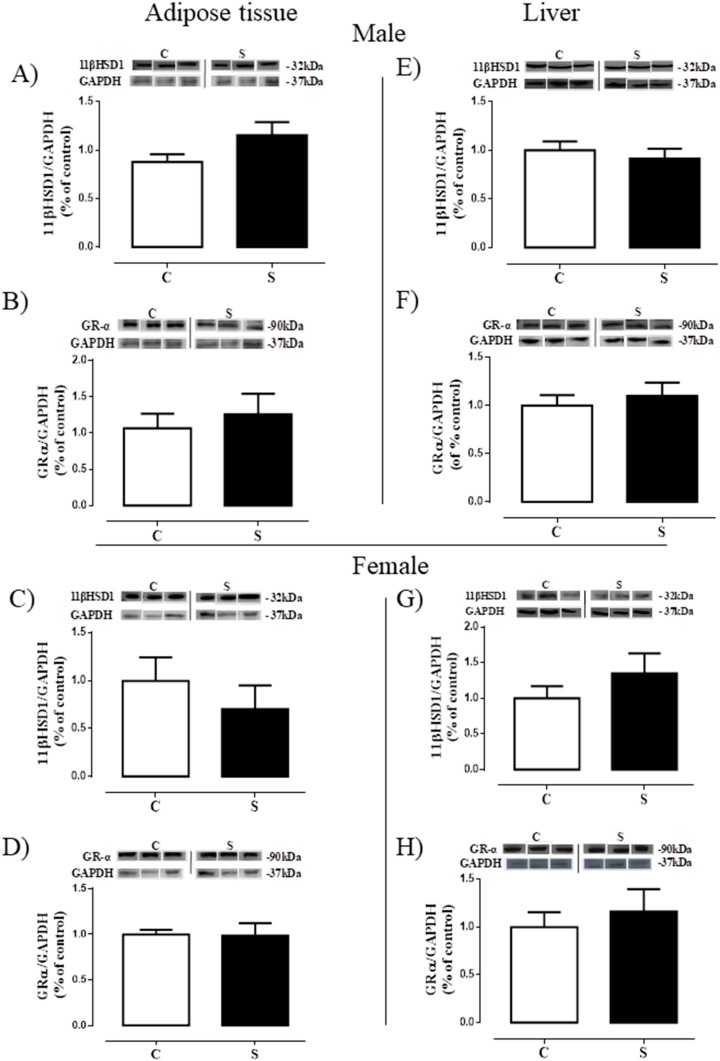
Glucocorticoid metabolism and action of adult rats programmed by smoke exposure during the lactation period. In the adipose tissue: 11β-hydroxysteroid dehydrogenase type 1 (11β-HSD1; **A**—male; **C**—female) and glucocorticoid receptor alpha (GRα; **B**—male; **D**—female); In the liver: 11β-HSD1 (**E**—male; **G**—female) and GRα (**F**—male; **H**—female). Groups: C—CONTROL and S—SMOKE. Values are given as means ± S.E.M. of 5–7 rats/group.

Figure 4 depicts the content of the enzyme that generates corticosterone (11β-HSD1) and the glucocorticoid receptor (GRα) in the VAT and liver. There were no differences between S and C groups in the adult offspring of both sexes (males: Figure 4A,B,E,F; females: Figure 4C,D,G,H) concerning these two glucocorticoids biomarkers on both tissues.

### 2.4. Tissue Metabolism and Serum Levels of 25 (OH) D

**Figure 5:**
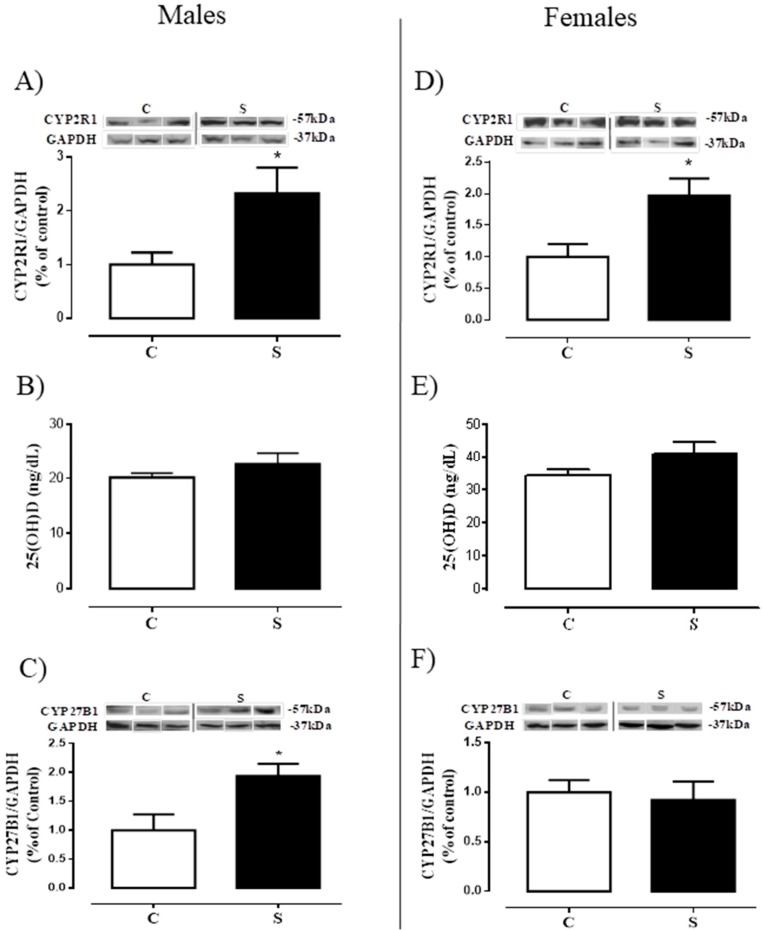
Vitamin D status of adult rats programmed by smoke exposure during the lactation period. Hepatic 25-hydroxylase (CYP2R1; **A**—male; **D**—female); Plasma 25-hydroxyvitamin D (25(OH)D) (**B**—male; **E**—female); Visceral fat 1α-hydroxylase (CYP27B1; **C**—male; **F**—female). Groups: C—CONTROL and S—SMOKE. Values are given as means ± S.E.M. of 5–10 rats/group. * *p* < 0.05 significant difference between S vs. C.

Figure 5 shows: (a) the content of the enzyme that generates calcidiol (25-hydroxylase) in the liver, (b) the calcidiol levels in the serum, and c) the content of the enzyme that activates the calcitriol in the VAT (1α-hydroxylase). Male offspring of the S group had higher 25-hydroxylase protein expression (+1.3-fold vs. C, *p* < 0.05, Figure 5A), unchanged 25 (OH) D levels (Figure 5B), and higher 1α-hydroxylase (+94% vs. C, *p* < 0.05, Figure 5C). Females of the S group had higher 25-hydroxylase in the liver (+97% vs. C, *p* < 0.05, Figure 5D), but no alterations in serum vitamin D and VAT 1α-hydroxylase (Figure 5E,F).

### 2.5. Lipogenesis in Visceral Adipose Tissue

**Figure 6:**
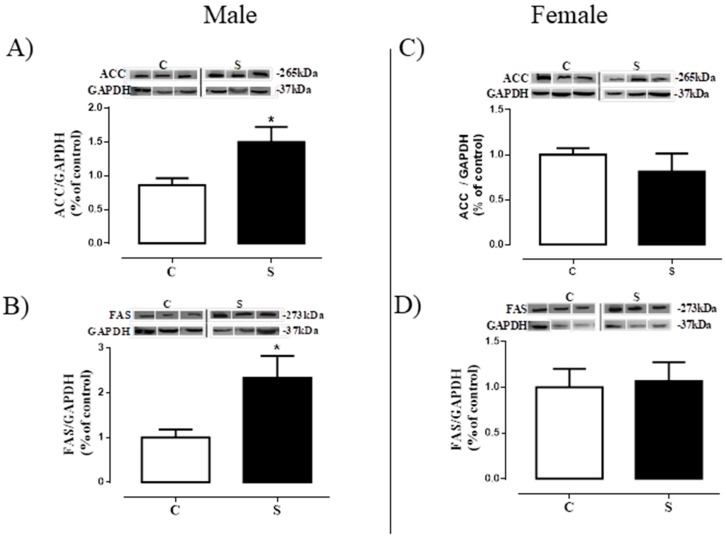
Lipogenesis in visceral adipose tissue of adult rats programmed by smoke exposure during the lactation period. Acetyl-CoA carboxylase (ACC; **A**—male; **C**—female); fatty acid synthase (FAS; **B**—male; **D**—female). Groups: C—CONTROL and S—SMOKE. Values are given as means ± S.E.M. of 4–7 rats/group. * *p* < 0.05 significant difference between S vs. C.

The contents of the proteins related to lipogenesis (ACC and FAS) are shown in Figure 6. We found higher ACC (+1.0-fold vs. male C *p* < 0.05, Figure 6A) and FAS (+1.3-fold vs. male C *p* < 0.05, Figure 6B) protein contents in the males of the S group when compared to controls. Females did not show differences between groups regarding ACC and FAS in the VAT (Figure 6C,D, respectively).

### 2.6. Lipogenesis in Liver

**Figure 7:**
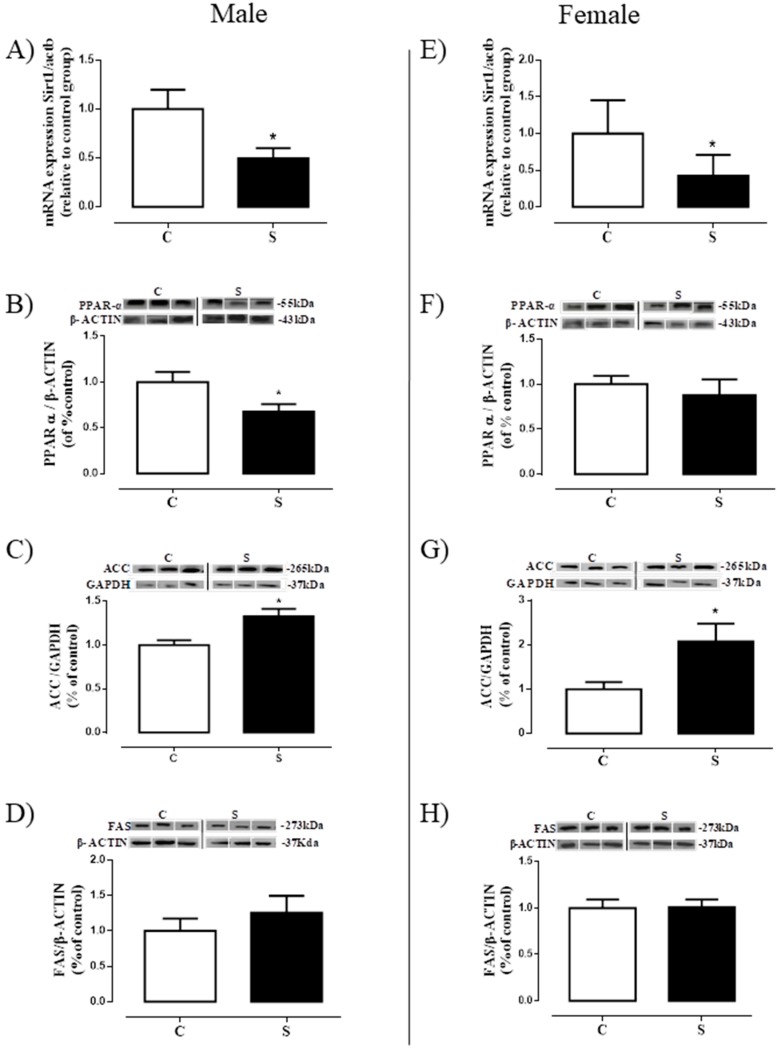
Lipogenesis in the liver of adult rats programmed by smoke exposure during the lactation period. Sirtuin-1 mRNA expression (Sirt1; **A**—male; **E**—female); peroxisome proliferator-activated receptor alpha (PPAR-α; **B**—male; **F**—female); acetyl-CoA carboxylase (ACC; **C**—male; **G**—female); fatty acid synthase (FAS; **D**—male; **H**—female). Groups: C—CONTROL and S—SMOKE. Values are given as means ± S.E.M. of 5–7 rats/group. * *p* < 0.05 significant difference between S vs. C.

S male animals showed reduced Sirt1 mRNA expression (−50% vs. C, *p* < 0.05, Figure 7A) and PPARα content (−32,3% vs. C, *p* < 0.05, Figure 7B), but higher ACC content (+33% vs. C, *p* < 0.05, Figure 7C) in the liver, with no changes in FAS protein expression (Figure 7D). Similarly, S females had reduced hepatic Sirt1 mRNA expression (−58% vs. C, *p* < 0.05, Figure 7E) and higher ACC content (+1.1-fold vs. C, *p* < 0.05, Figure 7G). However, no differences were found in the protein content of the PPARα (Figure 7F) and FAS (Figure 7H). 

## 3. Discussion 

In the present study, adult animals that were exposed to cigarette smoke during their breastfeeding period showed changes in serum corticosterone and tissue metabolism of vitamin D that may contribute to adipose tissue and liver dysfunction, although the morphology of both tissues was normal. Sexual dimorphisms were observed regarding circulating hormones and metabolic changes at the tissue level.

Recently, in an animal model of exposure to neonatal cigarette smoke, Lisboa et al. (2017) showed that adult animals of both sexes present increased abdominal adiposity. In the current study, these results were also observed [17]. However, when evaluating the morphology of VAT (retroperitoneal depot) and liver in these animals, surprisingly, no alteration of area or lipid accumulation was observed. Our study corroborates that of Zinkhan et al. (2014), in which exposure to smoke during pregnancy also increased adiposity in adult males without changes in adipocyte area [45]. Thus, here we suggest that the increase in adiposity observed in adult animals may be due to an increase in adipogenesis, indicating that this obesity is hyperplastic, not hypertrophic. We also did not detect changes in the contents of cholesterol and triglyceride in the liver, despite the several changes that were observed in lipogenic markers in the liver. In the literature, there are no data on the morphology of the hepatic tissue of animals exposed to cigarette smoke during the lactation period. In adolescent mice, chronic exposure to cigarette smoke does not alter the morphology of this tissue or the expression of genes that regulate lipid metabolism, inflammatory markers, and genes related to hepatic fibrosis [46].

The role of glucocorticoids as regulators of lipid accumulation is already well established; it acts by stimulating the adipocyte differentiation as well as the distribution of body fat [47]. In the current study, we observed alterations in corticosteronemia, with males showing a reduction and females an increase, which is indicative of a sexual dimorphism regarding tobacco smoke action regarding this parameter. To better understand this finding, we investigated MC2R in the adrenal, and glucocorticoid tissue metabolism (11β-HSD1 and GRα) in the VAT and liver. An increase of MC2R was observed in both sexes, without alterations in either GRα or 11β-HSD1 contents in the two tissues. This finding indicates that some alteration in the HPA (hypothalamus-pituitary-adrenal) axis secretion is present. The reduction in corticosteronemia seen in males may be due to primary adrenal hypofunction at adulthood, characterized by increased CRH-ACTH, and may be related to the hypercorticosteronemia of these animals during early life, as previously demonstrated [16]. An increase in ACTH secretion by the pituitary gland could overstimulate its receptors in the zona fasciculata of the adrenal gland, since it has been described that MC2R shows the unusual characteristic of being up-regulated by its ligand [48,49]. Conversely, females exposed to smoke were programmed for hypercorticosteronemia with increased MC2R, indicating a possible Cushing’s disease (ACTH dependent). Contrasting with males, females at the beginning of life did not present alterations in corticosteronemia [17]. Thus, we suggest that females exposed to smoke appear to be protected from its immediate effects, but not from adrenal function programming, by some still unknown mechanism. Another possibility is that cigarette smoke may directly increase CRH-ACTH axis or MC2R. However, Zinkhan et al. [45] demonstrated that intrauterine exposure to tobacco causes hypercorticosteronemia in the offspring of adult males (60 days), while hypocorticosteronemia is observed in females. The main difference between the aforementioned study and our present one is the period of smoke imprinting (gestation vs. lactation).

Some studies have demonstrated additional bone actions of vitamin D; among them is its action on lipid metabolism, which may directly influence the development of obesity [27,30]. However, the relationship between vitamin D and obesity is still quite controversial. Obese human subjects show reduced serum vitamin D levels [50,51], whereas in obese rodents, no change in serum levels was found [46]. In different experimental models of programming for obesity, an increase in 25 (OH) D concentrations was observed. Thus, it is possible that both the deficiency of and the resistance to this hormone are involved in the obesogenesis. For this reason, we evaluated the vitamin D status of adult animals programmed by the direct exposure to cigarette smoke during the lactation period. Despite no alteration in 25 (OH) D serum levels, both sexes showed increased hepatic 25-hydroxylase. It is possible that cigarette-smoke compounds may affect the absorption of vitamin D precursors, but the increase in liver 25-hydroxylase corrects the calcidiol serum levels. Also, this hormone may be highly activated to 1.25 (OH) D in the kidney by 1α-hydroxylase or inactivated to 24.25 (OH) D by 24 hydroxylase. In obesity, it has been suggested that the reduction in circulating vitamin D levels is due to a greater sequestration by adipocytes. The male offspring showed higher 1α-hydroxylase in the visceral adipocyte, which may indicate a greater uptake, and consequently, a higher local vitamin D formation. The increase in VAT 1α-hydroxylase has already been observed in other models of obesity programming, i.e., those in which a reduction of VDR is also present, suggesting a resistance to vitamin D action [37,52]. Thus, since vitamin D has anti-adipogenic effects [31,32,33], it is possible that our animals, which were programmed by the exposure to tobacco during the lactation period, developed resistance to vitamin D in the adipocyte, which contributes to their hyperplasia. Unlike males, the metabolism of vitamin D in the adipocyte of females does not seem to contribute to the increase in their adipocyte mass, characterizing a sex-dependent effect.

In order to understand the increase in central adiposity in our model, we analyzed the lipid metabolism in both sexes and, curiously, we observed alterations only in males, with increases in ACC and FAS, enzymes that are responsible for the synthesis of triglycerides and fat acids [53,54]. The increase in triglycerides in the adipose tissue is indicative of cellular dysfunction, and may trigger cellular apoptosis [54]. The results in the male adipose tissue suggest an increase in triacylglycerol production and, as a consequence, an increase in central adiposity. As females do not have alterations in these enzymes, we suggest that this mechanism is sex-dependent. Our results corroborate the study of Fan et al. (2016), which used a model of exposure of mothers dams and pups to nicotine during the prenatal and postnatal periods. They demonstrated increased central adiposity and FAS in males, while females also showed increased central adiposity but without alterations in FAS activity, suggesting a deleterious effect of nicotine programming on lipogenesis, with sex-related differences. While we realize that cigarette smoke has thousands of substances, we think that the changes described in our study are directly related to the effects of nicotine, mainly due to the results observed by Fan et al. [55].

Although we did not observe important alterations in the liver tissue under optical microscopy, we analyzed transcription factors and proteins involved in lipid metabolism: Animals presented an imbalance in hepatic lipid metabolism, with reduced markers related to lipid-β-oxidation and increased markers related to lipogenesis. The relationship between smoking, mainly through the actions of nicotine, and sirtuin, a histone deacetylase (HDAC), has already been established. For example, exposure to cigarette smoke reduces Sirt1 levels in vitro and in vivo, with associated increases in the release of proinflammatory cytokines [44,56]. In addition, there is a direct relationship between sirtuins and PPARs: Sirt1 activates PPARα through the deacetylation of PGC-1α [57,58], inhibiting its transcription. In the present study, males had a reduction in both Sirt1 and PPARα, which is indicative of the mechanism through which lipid accumulation occurs in the liver, and that may contribute to the genesis of hepatic steatosis. Corroborating this idea of hepatic dysfunction, we observed increased ACC in the male offspring. Regarding females, we observed a similar profile in the analyzed markers when compared to males, although liver PPARα was unaffected. It is important to emphasize that they also show increased ACC.

## 4. Materials and Methods

### 4.1. Animal Research

This study was approved by the Animal Care and Use Committee of the Biology Institute of the State University of Rio de Janeiro (CEUA/018/2014; 28 January 2014) and was performed in accordance with the principles adopted by Brazilian Law no. 11.794/2008 (8 October 2008). Wistar rats were appropriately allocated in a temperature-controlled room (23–24 °C) with artificial light and dark cycles (lights on: 07.00 h; lights off: 19.00 h).

For mating, 3-month-old virgin female rats were kept with male rats in a 2:1 proportion for 1 week. After mating, pregnant females were placed in individual cages with free access to water and standard chow (Nuvilab, Sogorb, São Paulo, SP, Brazil) during gestation and breastfeeding. At birth, litters were adjusted to 6 rats (3 females and 3 males) per dam.

#### Experimental Model of Direct Tobacco Smoke Exposure during Suckling Period

Three days after birth, rat dams and their litters were randomly assigned into one of the following groups: (a) SMOKE (S, *n* = 10): dams and offspring of both sexes were placed into a smoking machine (TE-10, Teague Enterprises, CA, USA), 4 1-h exposures times per day (1 h for each exposure). The tobacco smoke used was obtained from burning one cigarette type 3R4F at a time (nicotine = 0.73 mg/cigt; Reference Cigarette Program, University of Kentucky, Lexington, KY, USA) [16,17,59]. (b) CONTROL (C, *n* = 10): dams and offspring of both sexes were exposed to ambient air in a chamber that was similar to the one used for tobacco smoke exposure. The experimental cigarettes used in the present study produce cotinine levels in dams and pups at the end of lactation period of, respectively, 105 ng/mL and 145 ng/mL [16], which are similar to those observed in typical (heavy) smokers [60]. After weaning, body mass and food intake were monitored every 4 days until 180 days.

### 4.2. Histology

Fragments of the visceral (retroperitoneal) adipose tissue (retroperitoneal) and liver obtained from all lobes were fixed in freshly prepared fixative (1.27 mol/L formaldehyde, 0.1 mol/L phosphate buffer, pH 7.2) for 48 h at room temperature. Histological processing was carried out with alcohol baths, xylol and paraffin. Tissues were embedded in Paraplast Plus (Sigma-Aldrich, St. Louis, MO, USA) to obtain random cuts with a thickness of 5 μm. These were then stained with hematoxylin-eosin (HE). Adipocyte size was determined using the software Image-Pro Plus, version 5.0 (Media Cybernetics, Silverspring, MD, USA), with the use of randomly acquired digital images (TIFF format, 36-bit color, 1360 × 1024 pixels, 40× magnification) using an Olympus BX40 microscope with an Olympus DP71 camera (Olympus, Tokyo, Japan). A total of 50 adipocytes per animal (*n* = 8 offspring per sex/group) were measured, totaling 400 adipocytes per group. 

For the liver (*n* = 5–6 offspring per sex/group), the images were acquired with a 60× magnification (TIFF format, 36-bit color, 1360 × 1024 pixels). The method used for the stereological evaluation was the point counting system [61]. The magnitude of steatosis, based on Brunt’s classification with modifications for rats, was evaluated by two blind researchers. Thus, steatosis was graded as follows: 0 (none to 5% of hepatocytes affected), 1 (5% to 30% of hepatocytes affected), 2 (30% to 60% of hepatocytes affected), and 3 (60% of hepatocytes affected). The representative images were acquired with a 60× magnification (TIFF format, 36-bit color, 1360 × 1024 pixels). 

### 4.3. Hepatic Determination of Triglyceride and Cholesterol Contents

Liver samples (50 mg) were homogenized in 1 mL isopropanol (Vetec, Rio de Janeiro, Brazil) and centrifuged (2000× *g*, 10 min, 4 °C). We used *n* = 10 offspring per sex/group. The total triglyceride and cholesterol levels were measured using a colorimetric method with a commercial kit (Bioclin, Belo Horizonte, Brazil), as previously reported [62].

### 4.4. Serum Hormones Analysis—Radioimmunoassay (RIA) and Electrochemiluminescence Immunoassay (ECLIA) 

In both assays, samples were analyzed in duplicate (*n* = 10 offspring per sex per group). Corticosterone levels were evaluated by RIA kit (ImmuChem TM 125I, coated tube, ICN Biomedicals Inc., New York, NY, USA) following the manufacturer’s instructions. The 25(OH)D was quantified using a commercial ECLIA kit (Elecsys Vitamin D total assay, Roche Diagnostics GmbH, Mannheim, Germany) in accordance with the manufacturer’s instructions. This hormone is generally used to determine overall vitamin D status [63].

### 4.5. Western Blotting

In the visceral adipose tissue and liver, we evaluated the protein contents of the glucocorticoid receptor α (GRα), 11β-hydroxysteroid dehydrogenase type 1 (11β-HSD1), acetyl-CoA carboxylase (ACC) and fatty acid synthase (FAS). In the visceral adipose tissue, we also determined the 1α-hydroxylase (CYP27B1) content. In the liver, we also further measured vitamin D 25-hydroxylase (CYP2R1) and peroxisome proliferator-activated receptor alpha (PPAR-α) contents. In the adrenal gland, we evaluated the melanocortin receptor (MC2R) content, which is an ACTH receptor. In all tissues, we used *n* = 7 offspring per sex/group.

Adipose tissues were homogenized in liquid nitrogen with RIPA buffer + deoxycholate 0.5% (50 mM Tris HCL (pH 8.0), 150 mM NaCl, 0.5% deoxycholate, 1% NP-40, 0.1% SDS) with protease inhibitor cocktail (F. Hoffmann-La Roche, Basel, CH). Livers were homogenized in ice-cold RIPA buffer (50 mM Tris–HCl (pH 7.4), 1% NP-40, 150 mM NaCl, 1 mM EDTA, 1 mM PMSF, 1 mM Na3VO4, 1 mM NaF) with protease inhibitor cocktail (F. Hoffmann-La Roche Ltd.a., Basel, CH). Samples were then centrifuged for 25 min (18,506× *g*, 4 °C). Adrenals were homogenized with sodium phosphate buffer 0.1 M (pH 7.4%) with protease inhibitor cocktail (F. Hoffmann-La Roche., Basel, Switzerland). Protein concentrations were determined using the Pierce BCA Protein Assay Kit (Thermo Fisher Scientific, Waltham, MA, USA). Homogenates were analyzed by SDS-PAGE using 20 µg total protein. Samples were electroblotted into nitrocellulose membranes (Hybond ECL; Amersham Pharmacia Biotech, London, UK) and then incubated with Tris-buffered saline (TBS) containing 5% albumin for 45 min, except for the membrane with 11β-HSD1, which was incubated with 0.5% albumin for 90 min. Following that, membranes were washed with TBS and incubated overnight at 4 °C with a specific primary antibody: anti-11β-HSD1 (1:200; Cell Signaling Technology Inc. Danvers, MA, USA), anti-GRα (1:500, Santa Cruz Biotechnology, Inc., Santa Cruz, CA, USA), anti-ACC (1:500, Cell Signaling Technology, Inc. Danvers), anti-FAS (1:800, Cell Signaling Technology, Inc. Danvers), anti-PPAR-α (1:500; Santa Cruz Biotechnology, Inc., Santa Cruz, CA, USA), anti-CYP27B1 (1:500; Santa Cruz Biotechnology, Inc.), anti-CYP2R1 (1:200; Santa Cruz Biotechnology, Inc.), anti-MC2R (1:500; EMD Millipore Corporation, Temecula, CA, USA), anti-β-actin (1:500, Sigma Aldrich, Invitrogen Corporation, Carlsbad, CA, USA), and anti-GAPDH (1:1000; Cell Signaling Technology, Inc., Danvers, MA, USA). Membranes were then washed three times with Tween-TBS (0.1%) and incubated for 1 h with an appropriate secondary antibody conjugated with biotin (1:7000 and 1:10,000, anti-rabbit, anti-goat or anti-mouse from Sigma-Aldrich, Invitrogen Corporation, Carlsbad, CA, USA) at room temperature. Then, the membranes were washed again three times with Tween-TBS (0.1%) and incubated with streptavidin-conjugated horseradish peroxidase (Caltag Laboratories, Burlingame, CA, USA). The protein bands were visualized by chemiluminescence (Kit ECL plus, Amersham Biosciences, London, UK) followed by exposure to ImageQuant LAS (GE Healthcare, Buckinghamshire, UK). The area and density of the bands were quantified using the Image J software (Wayne Rasband National Institute of Health, Bethesda, MA, USA) and normalized against the bands obtained for β-actin. Results were expressed as percentages (%) of the control group (SD).

### 4.6. Sirtuin 1 (Sirt1) mRNA Expression in the Liver

Tissues were stored in RNAlater (Qiagen, Valencia, CA, USA) at −80°C, to avoid RNA degradation, until the moment of mRNA extraction. Total RNA from the same amount of each sample (50 mg) was extracted with TRIzol reagent (Invitrogen, Carlsbad, CA, USA), under RNAse-free conditions. Before cDNA confection, the quantity and the quality of the RNA in each sample were evaluated using the NanoVueTMPlus Spectrophotometer (GE Healthcare, Buckinghamshire, UK). Only the samples that had 260/280 ratio between 1.8–2.0 and the 260/230 ratio between 1.8–2.2 were used on in the RT-PCR assays. The cDNA was constructed from 1 µg of total RNA using the Moloney Murine Leukemia Virus Reverse Transcriptase (M-MLV RT) for RT-PCR and Oligo(dT)15 Primer (Promega, Madison, WI, USA). Before the RT-PCR assay, the amplification efficiency of each probe was analyzed by the standard curve and the appropriate threshold and cDNA dilution were determined. The mRNA expression was analyzed by RT-PCR carried out in duplicate for each sample using an Applied Biosystems 7500 Real-Time PCR System (Applied BioSystems, Foster City, CA, USA). To ensure the absence of genomic DNA contamination, a minus RT reaction (RT-) was performed in all RT-PCR assays: no amplification product (Cq value) was detected in any of the RT- control reactions. The expression of Sirt1 (*Rn01428096_m1*) mRNA was quantified using TaqMan^®^ Fast Universal PCR Master Mix (2×) AmpErase^®^ UNG (Catalog #: 4324018) (Applied Biosystems^®^, Foster City, CA, USA) in accordance with the recommendations of the manufacturer. A co-amplification of Actin-beta gene (Assay ID: *Rn00667869_m1*) was also performed in all samples. This gene was chosen as a reference since both the Control and Smoke groups exhibited similar Cq values. Results were analyzed using the ΔΔ*C*_T_ method. We used from 5 to 7 offspring per sex/group.

### 4.7. Statistical Analysis

The statistical analyses were carried out using the Graph Pad Prism 5.0 for Windows statistical software (GraphPad Software, La Jolla, CA, USA). Comparisons between groups were performed using Student’s unpaired *t*-test because the metabolic programming effect was evaluated separately for males and females. Data are shown as means and standard error of the mean (S.E.M.). Differences were considered significant at *p* < 0.05.

## 5. Conclusions

Here, we evidenced that the hormonal and functional outcomes at adulthood of exposure to cigarette smoke exclusively during the lactation period are sex-dependent, being less severe in females. We suggest that sex steroid hormones may influence these differences in response: While testosterone enhances the deleterious effects of programming via exposure to cigarette smoke, estrogen may actually protect females from this endocrine disruptor in order to ensure normal reproduction.

## Figures and Tables

**Table 1:** Degree of liver steatosis of adult offspring programmed by smoke exposure.

Degree of Steatosis	MALES		FEMALES	
	C	S	C	S
Degree 0	100%	100%	100%	100%
Degree 1	0%	0%	0%	0%
Degree 2	0%	0%	0%	0%
Degree 3	0%	0%	0%	0%

Groups: C—CONTROL and S–SMOKE; *n* = 5–6 per group.

**Table 2:** Liver lipid levels of adult offspring programmed by smoke exposure.

Liver Lipids	MALES	
	C	S
Cholesterol Content (mg/dL)	1.95 ± 0.07	1.89 ± 0.04
Triglyceride Content (mg/dL)	2.21 ± 1.12	2.41 ± 0.05
	FEMALES	
	C	S
Cholesterol Content (mg/dL)	1.85 ± 0.04	1.90 ± 0.05
Triglyceride Content (mg/dL)	1.59 ± 0.04	1.65 ± 0.05

Groups: C—CONTROL and S—SMOKE. Results expressed as mean ± SEM; *n* = 10.
